# 900. Evaluation of automated blood culture results and subsequent pharmacist intervention on time to targeted therapy in patients with bacteremia

**DOI:** 10.1093/ofid/ofac492.745

**Published:** 2022-12-15

**Authors:** Jessica Spangler, Andrew Gentry, Anastasia Bilinskaya

**Affiliations:** Backus Hospital, Norwich, Connecticut; Backus Hospital, Norwich, Connecticut; Hartford Healthcare, Hartford, Connecticut

## Abstract

**Background:**

Bloodstream infections (BSIs) are associated with a high mortality rate and timely administration of empiric broad-spectrum antibiotics is critical. However, prolonged exposure increases the risk of colonization and subsequent infection with resistant bacteria. The use of rapid diagnostics and prospective audit and feedback allows for decreased time to targeted therapy and development of resistance.

**Methods:**

This quasi-experimental study aimed to evaluate changes in time to targeted antibiotic therapy before and after implementation of a real-time pharmacist notification for bacteremic patients. The pre- and post- intervention periods were 9/1/19–12/31/19 and 9/1/21– 12/31/21, respectively. Bacteremic patients that were identified via automated Bruker’s MBT Sepsityper IVD kit reports and admitted for ≥48 hours were included in the study. Patients were excluded if they had ≥ 1 BSI or polymicrobial BSI during hospitalization, treatment of a concurrent infection, or transitioned to hospice. The primary outcome was change in time to targeted antibiotic therapy. Secondary outcomes included change in broad-spectrum antibiotic days of therapy, total duration therapy, hospital length of stay, and in-hospital all-cause mortality.

**Results:**

A total of 132 charts were reviewed, 83 of which were included, with 47 in the pre- and 36 in the post-intervention group. The most common isolates in the study across both groups was E. coli (36.1%), Klebsiella spp. (15.7%), MSSA (15.7%) and MRSA (8.4%). A trend towards decreased time to targeted therapy was seen in the post-intervention group (5.4 hours vs 33.0 hours; p = 0.150). Median duration of anti-MRSA therapy was reduced from 49.4 hours in the pre-intervention group to 27.92 hours in the post-intervention group (p=0.035). There was no significant difference in total duration of therapy, days of anti-pseudomonal therapy, length of stay, or in-hospital all-cause mortality. Of the 22 pharmacist interventions, 18 (81.8%) were accepted. The most common pharmacist intervention was therapy de-escalation (72.7%).

**Conclusion:**

Automated rapid blood culture diagnostic results used in conjunction with real-time notifications to pharmacists, further decreased time to targeted therapy and significantly reduced duration of anti-MRSA therapy.

**Disclosures:**

**All Authors**: No reported disclosures.

